# Molecular and Physiological Aspects of SARS-CoV-2 Infection in Women and Pregnancy

**DOI:** 10.3389/fgwh.2022.756362

**Published:** 2022-02-24

**Authors:** Anna Liu, Janet Raja xavier, Yogesh Singh, Sara Y. Brucker, Madhuri S. Salker

**Affiliations:** ^1^Research Institute of Women's Health, Eberhard Karls University, Tübingen, Germany; ^2^Institute of Medical Genetics and Applied Genomics, Eberhard Karls University, Tübingen, Germany

**Keywords:** COVID-19, SARS-CoV-2, pregnancy, vertical transmission, decidualization, ACE2, women

## Abstract

Whilst scientific knowledge about SARS-CoV-2 and COVID-19 is rapidly increasing, much of the effects on pregnant women is still unknown. To accommodate pregnancy, the human endometrium must undergo a physiological transformation called decidualization. These changes encompass the remodeling of endometrial immune cells leading to immunotolerance of the semi-allogenic conceptus as well as defense against pathogens. The angiotensin converting enzyme 2 (ACE2) plays an important regulatory role in the renin-angiotensin-system (RAS) and has been shown to be protective against comorbidities known to worsen COVID-19 outcomes. Furthermore, ACE2 is also crucial for decidualization and thus for early gestation. An astounding gender difference has been found in COVID-19 with male patients presenting with more severe cases and higher mortality rates. This could be attributed to differences in sex chromosomes, hormone levels and behavior patterns. Despite profound changes in the female body during pregnancy, expectant mothers do not face worse outcomes compared with non-pregnant women. Whereas mother-to-child transmission through respiratory droplets during labor or in the postnatal period is known, another question of *in utero* transmission remains unanswered. Evidence of placental SARS-CoV-2 infection and expression of viral entry receptors at the maternal-fetal interface suggests the possibility of *in utero* transmission. SARS-CoV-2 can cause further harm through placental damage, maternal systemic inflammation, and hindered access to health care during the pandemic. More research on the effects of COVID-19 during early pregnancy as well as vaccination and treatment options for gravid patients is urgently needed.

## Introduction

Since its emergence in December 2019 in Wuhan, China, the Severe Acute Respiratory Syndrome Coronavirus 2 (SARS-CoV-2) has infected over 250 million people and caused more than 4.9 million deaths worldwide (as of October 2021) (1, 2). The disease caused by SARS-CoV-2 is termed Coronavirus Disease 2019 (COVID-19) and was declared a global pandemic in March 2020 (3). Whilst scientific knowledge about this disease is rapidly increasing, much of its effects on pregnant women is still unknown.

Pregnancy is a unique physiological state during which the female body undergoes profound transformations. The immune system is altered during pregnancy, resulting in immunotolerance of the semi-allogenic conceptus as well as protection of both mother and fetus against pathogens (4). Research indicates that during pregnancy, expectant mothers are more susceptible to some infectious diseases, such as influenza or Ebola (5, 6).

The aim of this review is to illustrate what is known about COVID-19 and how it affects pregnancy. First, changes in the human endometrium enabling embryo implantation and pregnancy will be discussed – a process coined decidualization. Special emphasis will be put on the endometrial immune microenvironment, angiotensin-converting enzyme 2 (ACE2) and transmembrane serine protease 2 (TMPRSS2). What follows is a brief overview of SARS-CoV-2 and of COVID-19. The review will further describe the gender differences found in COVID-19 and offer possible explanations. Lastly, what is known about implications of COVID-19 infection during pregnancy will be reviewed, with particular focus on the possibility of vertical transmission of SARS-CoV-2.

## The Human Endometrium and Decidualization

The human menstrual cycle is approximately 28 days long and can be divided into two phases: the follicular (proliferative) phase and the luteal (secretory) phase (7, 8). The start of each cycle is marked by the onset of menstruation (9). During the first phase, estrogen is produced by granulosa cells in the ovaries, which leads to thickening of the endometrium (7). This thickening is the result of proliferating epithelial and stromal cells, as well as angiogenesis (7, 10). Ovulation marks the start of the second phase, when the corpus luteum produces progesterone, further preparing the endometrium for the possibility of embryo implantation and pregnancy; a process known as decidualization (11, 12). In the case of no pregnancy, the corpus luteum deteriorates leading to a drop in progesterone levels, vasoconstriction in the endometrium with hypoxia and desquamation of the stratum functionalis (11–13).

**Figure 1:**
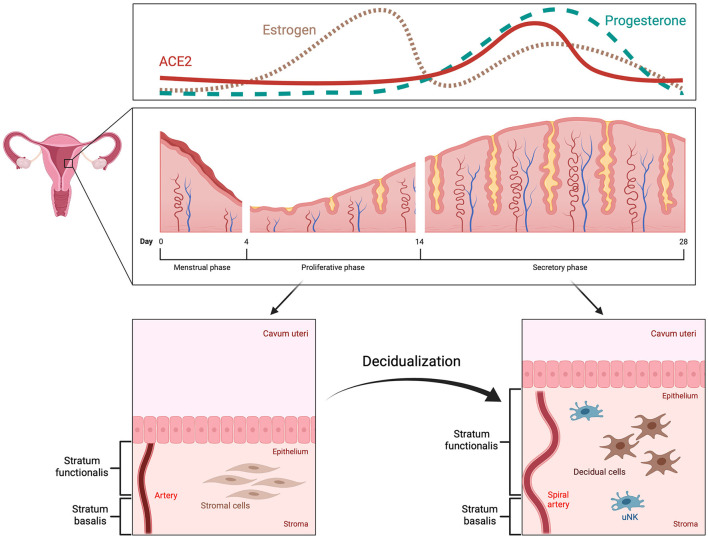
Menstrual cycle and decidualization. The human menstrual cycle repeats itself in 28-day intervals. The start is marked by the onset of menstruation. Subsequently, the endometrium enters the proliferative phase, during which it increases in thickness as a response to high estrogen levels (pink, dotted line). In the secretory phase, decidualization occurs with remodeling of spiral arteries, mesenchymal-epithelial transition of stromal cells and alterations in the endometrial immune system, e.g., increase in uterine natural killer cells. These changes are triggered and regulated by progesterone (green, dashed line) and mainly take place in the upper part of the endometrium, the stratum functionalis, which is also shed during menstruation. ACE2 expression is increased by decidualization in the secretory phase (red, solid line).

The process of decidualization involves the differentiation of endometrial stromal cells, which are of mesenchymal origin and resemble fibroblasts, into decidual cells, similar to epithelial cells (Figure 1) (14–16). During this mesenchymal-epithelial transition, the cells become larger and rounder with an expansion of the rough endoplasmic reticulum and the Golgi apparatus (11, 14). There is an increase in the number of nucleoli and an accumulation of lipid and glycogen droplets within the cytoplasm (11, 16). It was also shown that polyploidy is common among decidual cells, which might limit their lifespan but could benefit the growth of the embryo due to increased protein synthesis (17, 18). Decidual cells produce large quantities of prolactin and insulin-like growth factor binding protein-1, among others, which can also be used as *bona fide* markers for decidualization (11, 14).

The human endometrium is subject to cyclic transformations to provide an optimal environment for embryo implantation, however, the window of implantation is brief (19). The uterus is only receptive to a blastocyst during the limited duration of about 4 days, approximately 6 to 10 days after ovulation (20, 21). Not only does decidualization influence the timing of implantation but it also controls the extent of invasion by the embryo (22). Some studies even suggest that decidual cells are not passively invaded by the trophoblast but actively encapsulate the embryo (23–25). Moreover, the endometrium has the capability to sense the quality of the conceptus and makes a distinction between healthy and impaired embryos (26, 27). Therefore, the decidua promotes implantation of high-quality embryos while rejecting developmentally impaired ones through modulation of gene expression (26–28). Defective decidualization can lead to a plethora of pregnancy complications such as preeclampsia, preterm birth or even recurrent pregnancy loss, highlighting the importance of adequate decidualization in early pregnancy (15, 29).

## The Endometrial Immune Microenvironment

Changes in morphology and function are not solely limited to stromal cells. Remodeling of the extracellular matrix as well as cell-cell interactions play a crucial role in decidualization (30, 31). Since pregnancy requires a well-calibrated balance between immunological responsiveness and tolerance, immune cells are another relevant component of the decidua (32, 33). During early pregnancy, up to 40% of all cells within the decidual tissue are leukocytes, such as macrophages, T and B cells and, most prominently, uterine natural killer cells (uNK) (34). The latter sees an increase in number during decidualization and is most abundant in the vicinity of spiral arteries, endometrial glands and at the maternal-fetal interface (7, 35). Although their function is not completely clear, studies suggest that uNK are involved in remodeling of spiral arteries, clearance of senescent decidual cells, regulating maternal immune tolerance and defense against pathogens (15, 35, 36).

The maternal immune system is modulated during pregnancy, which is particularly meaningful when trying to understand the effects of COVID-19 on pregnancy and vice versa. The decidualized cells play an important role in providing immunotolerance toward the allogenic embryo by modulating the spectrum of immune cells at the maternal-fetal interface (32, 37). While there are plenty uNK cells (70% of total leukocytes) and macrophages (20–25%) in the decidual tissue, dendritic cells and B and T lymphocytes are rare (11, 32, 38). It has been shown that dendritic cells, which regularly trigger T cell reaction, are entrapped in the tissue through decidualization and that their density throughout the decidua is reduced (39). Due to this entrapment, the dendritic cells are ineffective in facilitating T cell activation, thus, lowering the chance of immunological attack on the fetus. Furthermore, decidual cells inhibit T cell proliferation, suppress inflammation and prompt apoptosis of activated T cells *via* the expression of Galectin-1, indoleamine-2,3-dioxygenase and FAS-ligand (40–42).

In summary, decidualization is part of the cyclic morphological alterations in the endometrium. This process is of utmost importance for embryo implantation and early pregnancy. Decidualization mainly encompasses modifications in endometrial stromal cells and is regulated predominantly by progesterone. Further, changes also occur in endometrial immune cells, and uNK cells are of particular interest, as they play an important role in endometrial remodeling during decidualization as well as immune tolerance and defense.

## ACE2 – Physiology and Role in Decidualization

**Figure 2:**
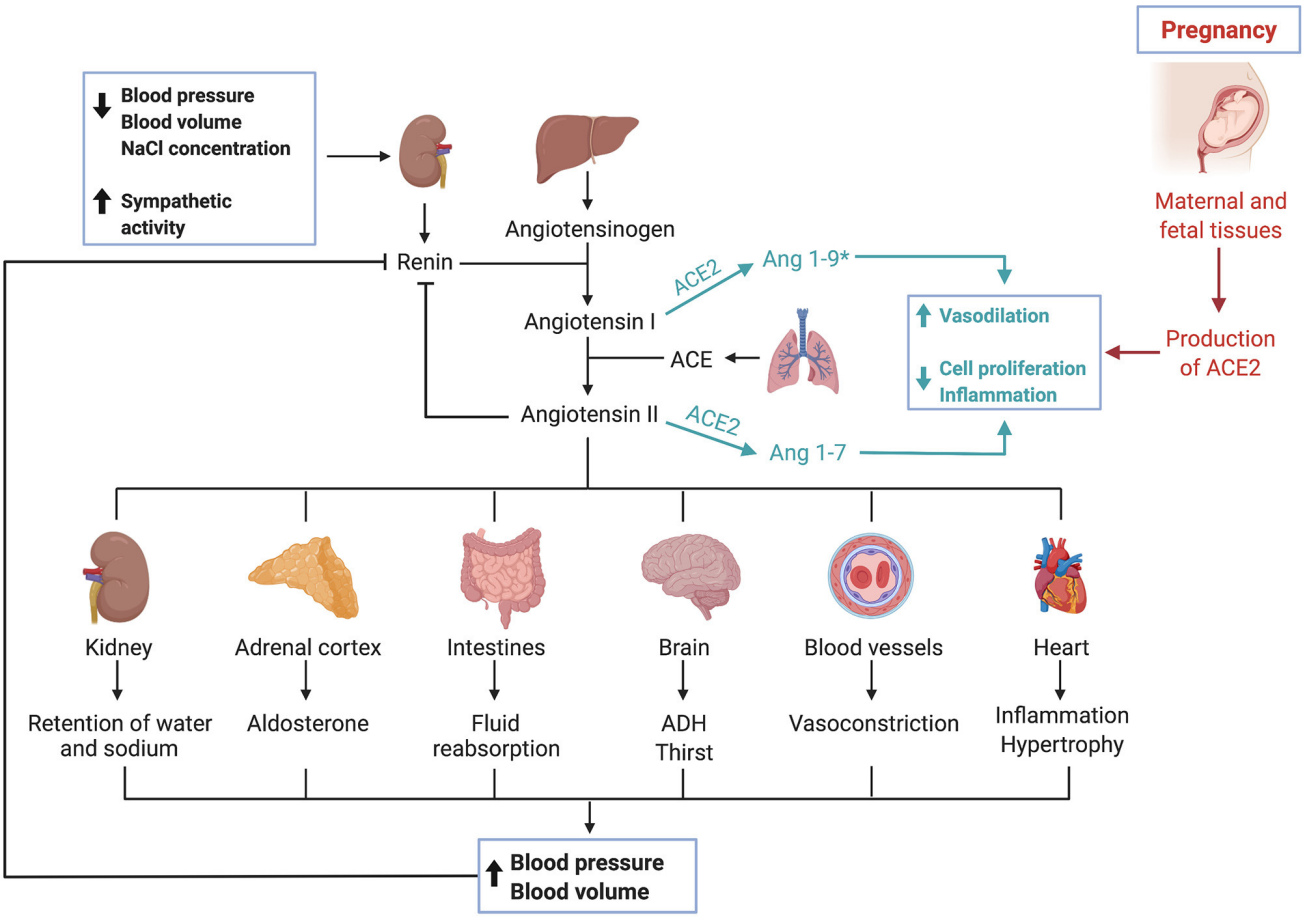
The renin-angiotensin-system and ACE2. Triggered by low blood pressure, low blood volume and low sodium levels as well as sympathetic activity, the kidney secretes renin. Renin, a protease, cleaves angiotensinogen, secreted by the liver, into angiotensin I. Angiotensin I is then converted by the angiotensin-converting enzyme, which can be found in membranes of endothelial cells most abundantly in the lungs and kidneys, into angiotensin II. Various effects are caused by angiotensin II, which ultimately result in an increase of blood pressure and volume. The angiotensin-converting enzyme 2 works as a counterbalance to ACE by cleaving angiotensin I and angiotensin II into Ang 1–9* and Ang 1–7, respectively. (*Ang1–9 is postulated to exert similar effects as Ang1–7, though current data is limited and needs further validation). Activation of this pathway leads to vasodilation, inhibits cell proliferation and has anti-inflammatory effects. During pregnancy, maternal and fetal tissues contribute to the production of ACE2, leading to systemic vasodilation.

The angiotensin-converting enzyme 2 (ACE2) is a typical zinc metallopeptidase that plays an important role in the renin-angiotensin system (RAS) (43). It is an integral membrane glycoprotein, consisting of 805 amino acids and containing a single catalytic domain (44). Major functions of ACE2 include converting angiotensin (Ang) I into Ang 1-9 and Ang II into Ang 1-7 (45, 46). ACE typically converts Ang I into Ang II, causing vasoconstriction, leading to inflammation and fibrosis, ACE2 can be seen as a counterbalance to ACE (44). Thus, ACE2 is a negative regulator of RAS and therefore crucial in regulating blood pressure, fluid and electrolyte balance (Figure 2) (47).

Despite being expressed ubiquitously in the human body, some tissues contain remarkably high amounts of ACE2 including the kidneys, heart, lungs, testes and intestines as well as endothelial and vascular smooth muscle cells (43, 48, 49). ACE2-expressing tissues are potential targets for SARS-CoV-2 (50).

Notably, ACE2 has been shown to be protective against heart failure, hypertension, diabetes, renal dysfunction and pulmonary diseases (51–55). These are also comorbidities that have been identified to worsen the outcome of COVID-19 patients, which might be linked to a dysregulation of RAS (43, 56, 57). After facilitating the entry of SARS-CoV-2 into the host cell, ACE2 expression is downregulated (58). Loss of ACE2 is initiated during virus infection since the SARS-CoV-2-ACE2-complex is internalized through endocytosis (59, 60). This depletion of ACE2 leads to a dysregulation of RAS, further aggravating harmful effects of COVID-19, such as acute respiratory distress syndrome (ARDS) and endothelial dysfunction (61–63).

A controversial debate over the continued usage of angiotensin II receptor blockers and ACE inhibitors among COVID-19 patients with preexisting hypertension arose after the ACE2 receptor became known as the entry factor for SARS-CoV-2 (64). Since these drugs were thought to increase the expression of ACE2, it was hypothesized that their application would lead to higher infection rates and more severe COVID-19 cases (65, 66). However, several studies found no significant difference between patients treated with or without ACE inhibitors and/or angiotensin II receptor blockers regarding the infection rate and COVID-19 outcome (67–70). Controversially, others even reported a lowered risk of SARS-CoV-2 infection or critical illness and death, respectively (71, 72). Notably, it was speculated that geographic and ethnic factors may influence the interaction between these drugs and COVID-19 (69, 71). Further studies are required to substantiate these findings.

The influence of gender and age on ACE2 expression is not fully understood yet. While several studies did not prove significant differences of ACE2 expression between young males and females (<55 years), it has been shown that the correlation between ACE2 and immune signatures in the lungs differ between the two sexes (49, 73–75). There have been contrasting results regarding the relation of ACE2 content to increasing age, with some finding an increase, a decrease, or no change at all (49, 73, 74). However, it has been suggested that steroid hormones may affect ACE2 activity with withdrawal of estrogen or testosterone causing an increase or a decrease, respectively (75).

Another important albeit overlooked function of ACE2 was illustrated in a recent study. Chadchan et al. found that the ACE2 protein is not only highly expressed in human endometrial stromal cells (HESCs), in particular during the secretory phase of the menstrual cycle, but it also increases significantly in stromal cells undergoing decidualization *in vitro* (76). They further observed that loss of ACE2 impeded decidualization (76). Additionally, Chadchan et al. described that ACE2 expression in the endometrium is induced by progesterone. Considering these results, it is plausible that ACE2 plays a vital role in decidualization of the human endometrium.

Furthermore, other studies have found that ACE2 and other components of RAS are expressed both in maternal and fetal tissues during pregnancy, suggesting their crucial role during implantation, vascular remodeling and labor (77–81). During pregnancy, the uterus and placenta contribute substantially to ACE2 production, thus causing a twofold increase in ACE2 activity with subsequent systemic vasodilation (82). The upregulation of RAS in the maternal decidua as well as in the endothelial and perivascular stromal cells during the first trimester of pregnancy coincides with spiral artery remodeling and angiogenesis (83). Dysregulation of uteroplacental RAS is reported to alter the tightly regulated maternal homeostasis causing pregnancy complications such as miscarriage, still birth and preeclampsia (84–86). It was also shown that plasma Ang 1-7, a product of ACE2, is elevated during healthy pregnancies and that preeclamptic mothers had lower levels of Ang 1-7 (87).

Remarkably, ACE2 is most abundant in the decidua in comparison with chorionic or amniotic tissues (77). Another compelling finding is that ACE2 expression is highest during early pregnancy and is negatively correlated with gestational age (88, 89). Moreover, fetal sex might affect maternal RAS and for instance, ACE2 mRNA levels were higher in decidual explants after 24h from women carrying a female fetus compared with those carrying a male fetus (90).

Briefly, ACE2 has essential functions for the RAS and contributes to the control of blood pressure as well as fluid and electrolyte homeostasis. ACE2 is expressed in various tissues throughout the human body and is protective against cardiovascular and respiratory diseases, among others. ACE2 is essential for decidualization and its production increases during pregnancy. Since ACE2 is also the entry receptor for SARS-CoV-2, its high expression in placental tissues has implications for pregnancies during COVID-19 infection, which will be covered in detail below.

## SARS-CoV-2 and COVID-19

SARS-CoV-2 belongs to the same genus betacoronavirus as SARS-CoV and MERS-CoV, which are all enveloped viruses with a single-stranded positive-sense RNA (91, 92). Although the origin of SARS-CoV-2 has not been fully clarified yet, it is most likely that it originated from bats, which are a natural reservoir for coronaviruses, and was passed on to humans *via* an intermediate host such as pangolins (93, 94).

**Figure 3:**
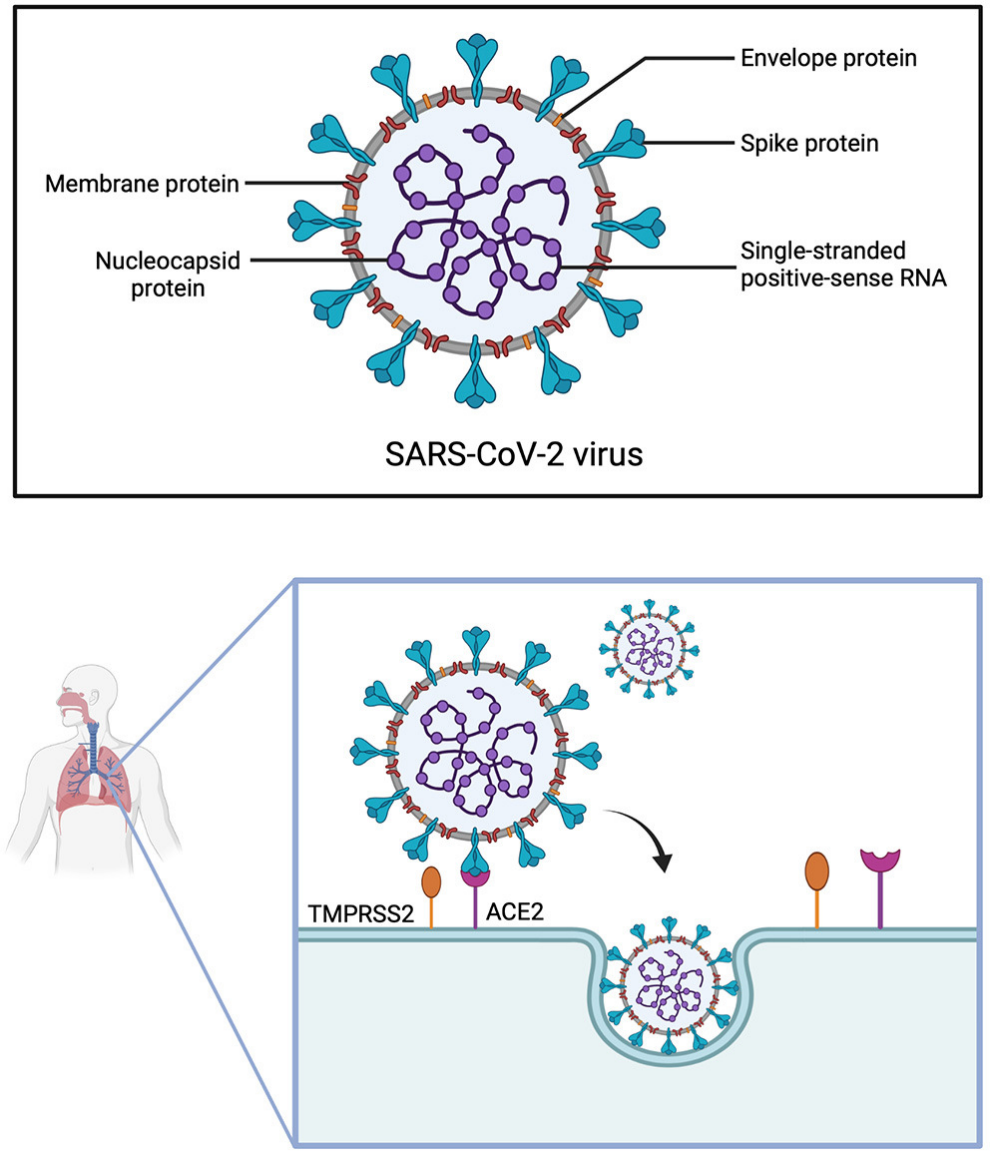
Structure of SARS-CoV-2 and cell entry. The Severe Acute Respiratory Syndrome Coronavirus 2 is made up of four structural proteins (envelope, membrane, spike and nucleocapsid protein) and has a single-stranded positive-sense RNA. For SARS-CoV-2 to infect cells of the respiratory tract, the spike protein first has to be cleaved by the transmembrane protease serine 2 (TMPRSS2), before it can interact with the angiotensin-converting enzyme 2 (ACE2) receptor. The virus then enters the cell through endocytosis.

SARS-CoV-2 consists of four structural proteins: spike (S), nucleocapsid (N), membrane (M) and envelope (E) protein (95). The S-protein is of utmost interest, as it facilitates virus entry into host cells (96, 97). Due to the similarity of their S-proteins, SARS-CoV and SARS-CoV-2 both utilize the cell surface receptor ACE2 for attachment and penetration of host cells (97). However, the receptor-binding domain (RBD) of the S-protein differs among SARS-CoV and SARS-CoV-2, resulting in higher binding affinity to ACE2 of the latter (98). A precondition for the interaction of SARS-CoV-2 with ACE2 is S-protein priming by host proteases, among which the most relevant seems to be transmembrane protease serine 2 (TMPRSS2) (Figure 3) (99).

The main route of transmission is through respiratory droplets, nevertheless, SARS-CoV-2 can be further spread *via* aerosols, direct and indirect contact, and feces (100–103). While it has been suggested that SARS-CoV-2 could be passed on *via* sexual contact and breastfeeding, more evidence is necessary (104, 105).

For COVID-19, the incubation period, defined as the time between infection and onset of symptoms, is approximately 5 days (106, 107). The basic reproduction number R_0_ of SARS-CoV-2 is estimated to lie between 2 and 3 with a peak viral load in the upper respiratory tract around symptom onset or shortly after (108–110). Not only can patients be infectious 1 to 3 days before any symptoms occur, but there have also been reports of asymptomatic transmission (110, 111).

The clinical presentation of COVID-19 varies greatly from asymptomatic and mild to critical and even fatal cases (112). Diagnosis is further impeded by unspecific symptoms, which resemble the clinical picture of the common cold, influenza or other respiratory diseases (113). The most common manifestations are fever and cough, which are present in the majority of the patients, followed by fatigue and shortness of breath (50, 92, 114). Anosmia, ageusia, myalgia and diarrhea are less frequent among COVID-19 patients (115, 116). Some symptoms, for instance fatigue or dyspnea, can persist despite microbiological recovery – a condition termed Long COVID (117, 118).

COVID-19 originally revealed itself through an outbreak of pneumonia (97). In severe cases, patients develop ARDS with hypoxia or even respiratory failure (119). Histopathological findings are diffuse alveolar damage with desquamation of pneumocytes, formation of hyaline membranes, edema and inflammatory infiltration by lymphocytes, as well as microvascular injury (120, 121). Correspondingly, chest computed tomography (CT) scans of COVID-19 patients commonly show bilateral distribution of ground-glass opacities with or without consolidations, “crazy paving” patterns and air bronchogram signs (122, 123).

COVID-19 not only involves the lungs and the respiratory tract, but also multiple organ systems (124). This includes cardiovascular (e.g. acute cardiac injury, myocarditis), gastrointestinal (e.g. nausea and vomiting, diarrhea), neurological (e.g. dizziness, stroke) and hematological manifestations (e.g. lymphocytopenia, thrombotic events, disseminated intravascular coagulation (DIC)) (124–128). A case point feature of COVID-19 is, that it triggers an extensive inflammatory response, the “cytokine storm”, which further aggravates damage done by the virus (129). A delay in immune response due to immune evasion of SARS-CoV-2 with consequentially unhindered virus replication is found in severe cases of COVID-19 (130–132). Virus-induced cell death prompts the recruitment of macrophages and neutrophils, followed by hyperinflammation (130). Subsequent tissue damage and multi-organ failure are the main cause of death in COVID-19 (133, 134).

The vast majority (80–90%) of COVID-19 cases are asymptomatic or mildly symptomatic, whereas among the critically ill the mortality rate is as high as 49% (112, 119, 135). The most important prognostic factor is age, with children mainly being asymptomatic or only exhibiting mild symptoms whereas elderly patients are at high risk for mortality (136–138). Other factors contributing to poor outcome are comorbidities such as hypertension, heart disease, diabetes mellitus, Chronic Obstructive Pulmonary Disease (COPD) and chronic renal disease (139–141). Furthermore, obesity and the male sex have been linked to increased severity of COVID-19 (141–143). Genetic factors also have an impact on COVID-19 infection and outcome with some studies suggesting links to HLA or ABO blood type (Table 1) (164–167).

**Table 1:** Risk factors for poor outcome in COVID-19.

**Risk factors**	**Association with COVID-19**
**Demographic characteristics**
Age	Children and younger people generally exhibit more asymptomatic or mild cases, whereas older patients are at higher risk for severe cases and death (136–138).
Sex	The male sex is associated with higher infection rates and worse outcomes compared with the female sex (138, 143–145).
Socioeconomic status	People with lower income are at increased risk of COVID-19 infection and higher mortality compared to those with higher income (146, 147).
**Comorbidities**
Hypertension	Most common comorbidity among COVID-19 patients, increases risk for poor outcome (138–140, 143)
Heart disease	Increases risk for poor outcome (138–141, 143)
Diabetes mellitus	Type 1 and type 2 diabetes as well as uncontrolled hyperglycemia increase the risk for poor COVID-19 outcome (139, 140, 143, 148–150). The association between high HbA_1c_ and increased mortality remains controversial (151–154). Especially for type 2 diabetes, use of insulin is linked to higher mortality (153, 155).
COPD	Increases risk for poor outcome (139, 140, 143, 156)
Chronic kidney disease	Increases risk for poor outcome (141, 157)
Obesity	Increases risk for poor outcome (140–143)
Cancer	Increases risk for poor outcome (139, 141, 158)
Chronic liver disease	Increases risk for poor outcome (159, 160)
**Genetic factors**
ACE2 (Angiotensin-converting enzyme 2)	Some polymorphisms increase susceptibility to SARS-CoV-2 (e.g., S19P, K26R, E23K), others hinder interactions between the spike protein and ACE2 (e.g., K31R, N33I, E329G) (161, 162).
TMPRSS2 (Transmembrane serine protease 2)	Some variants are linked to increased TMPRSS2 expression and higher susceptibility to SARS-CoV-2 (e.g., rs12329760) (163).
HLA (Human leukocyte antigen)	Variants encoding proteins with low binding affinity to SARS-CoV-2 (e.g., B^*^46:01, C^*^14:02) increase vulnerability, whereas variants encoding proteins with high binding affinity to SARS-CoV-2 (e.g., B^*^15:03, A^*^02:02) encourage immunity (164, 165).
ABO (blood groups)	Higher risk of infection for blood type A, lower risk for blood type O (166, 167).
**Lifestyle**
Smoking	Increases risk for poor outcome (156, 168)
Alcohol abuse	Uncertain, likely increases risk for poor outcome (169, 170)
Physical activity	Decreases risk for poor outcome (171, 172)

* *indicates Nomenclature of HLA alleles*.

## COVID-19 and Women

Historically, women were overlooked in biomedical research and the male model was seen as the “norm”. Women were and are still underrepresented in clinical trials due to the notion that studies could be complicated by the menstrual cycle and that a potential fetus could be harmed (173). This practice of exclusion from clinical trials is even more common for pregnant women (174). Indeed, sex discrepancy also applies to animal models with a male bias in the majority of research fields (175). The belief that male data can be simply extrapolated to women leads to inadequate treatment of female patients, such as wrong dosage of drugs or more severe side effects (173, 176).

An astounding gender difference has been found in COVID-19; in that whilst infection rates are similar in both sexes, men are prone to having more severe infection and higher mortality (two- to threefold) (138, 144, 145, 177). Likewise, this bias toward males was also present in the MERS outbreak in 2014 (178). However, this pattern was not as consistent in the previous SARS epidemic in 2002, with only one study showing a significant difference in case fatality ratio between men and women (179, 180). Controversially, another review even reported that mainly females were affected by SARS (181).

One possible rationale for the female advantage lies in the sex chromosomes (182, 183). Since women possess two X-chromosomes, one of the X-chromosomes is silenced to compensate gene dosage (184). Some genes escape inactivation resulting in differential expression between sexes (185). The gene of the SARS-CoV-2 receptor ACE2 is located on the X-chromosome and is further recognized as an escape gene (185, 186). This implies that women might be in a more favorable position of elevated ACE2 expression which counterbalances the downregulation of ACE2 upon SARS-CoV-2 infection and therefore protects from an overactive RAS. However, expression does not equal enzyme activity and as described above, sex differential expression of ACE2 is still controversial (187, 188). Notably, soluble ACE2 (sACE2), which is generated through shedding of membrane-bound ACE2, was found to be higher in men compared with women as well as postmenopausal compared with premenopausal women (189–191). As higher levels of sACE2 are correlated with cardiovascular disease and diabetes, known risk factors for more severe cases of COVID-19, this may contribute to the male disadvantage (192).

Another potential link between genetics and the purported reduced risk in females is the fact that the X-chromosome contains a great repertoire of immune-related genes (193). It is noteworthy that women generally mount a faster and stronger innate and adaptive immune response whereas men are subject to reduced immune response and higher pathogen load (194). X-linked genes are suggested to play a pivotal role in autoimmune diseases, which are characterized by a heightened immune response against the patient's own cells and primarily affect women (195, 196). Genes encoding for pattern recognition receptors (PRRs) have a vital function in the innate immune system and consequently are of special interest regarding the delayed immune response in COVID-19 (197). Among them, Toll-like receptor 7 (TLR7) is not only responsible for the recognition of single-stranded viral RNA in endosomes, but its gene is also located on the X-chromosome and known to escape silencing in immune cells (198–200). Furthermore, reaction to TLR7 stimulation also differs depending on sex as peripheral blood mononuclear cells from females produce more Interferon-α (IFN-α), eliciting an anti-viral response, whereas in males higher production of Interleukin-10 (IL-10), an immunosuppressive cytokine, is induced (201, 202). Considering that differences in the immune response between the sexes occurs across all age groups, it is perhaps plausible that sex chromosomes are part of the reason why females seem to clear pathogens faster and have less severe COVID-19 cases (203).

Endocrine factors are another conceivable explanation for sex disparities in COVID-19 outcomes as the mortality rate in postmenopausal women is higher than in premenopausal women (204, 205). Estrogen has potent immunomodulatory effects and estrogen receptors are expressed by several immune cells (206). At high concentrations, as found periovulatory or during pregnancy, estrogen has mainly anti-inflammatory effects, for instance decreasing levels of C-reactive protein (CRP), IL-6 and TNF-α (207–210). In contrast, estrogen triggers pro-inflammatory pathways at lower doses (211). Furthermore, estrogen has proven to reduce morbidity of influenza infection through modifying immune cell recruitment and cytokine production, as well as scaling down virus replication in females (212, 213). Despite less elderly women succumbing to COVID-19 in comparison to elderly men, climacteric women are still at higher risk of worse outcomes than their premenopausal counterparts, and futher, estradiol treatment was shown to improve survival (214, 215).

Certain comorbidities are known to negatively impact COVID-19 outcomes; it is noteworthy that the prevalence of hypertension, diabetes and COPD is lower in the female population (216–218). This can be attributed to women leading healthier lifestyles, such as less smoking, lower alcohol consumption and more physical activity (219–221). Nonetheless, biological factors might also have an impact on the development of the said comorbidities. Aside from ACE2 activity positively correlating with estrogen levels, protective effects of estrogen on atrial tissue through modulation of RAS and upregulation of ACE2 were observed (222, 223). Moreover, in a study on mice, obese females had higher adipose ACE2 activity and, unlike obese males, did not develop hypertension (224). When ovariectomized, female mice also showed reduced ACE2 activity and obesity-hypertension, which could be reversed through treatment with estrogen (224, 225). Akin to estrogen's shielding properties in hypertension, estrogen also guards premenopausal women from diabetes mellitus and estrogen replacement therapy in postmenopausal patients is beneficial for metabolic health (226, 227). In respect to COVID-19, it is noteworthy to point out that estrogen was shown to decrease ACE2 expression of differentiated airway epithelial cells, providing another clue for sex-difference in infection (228).

In summary, COVID-19 affects men with disproportionately higher infection rates, more severe cases, and higher mortality than women. This can be attributed to sex chromosomes, as the *ACE2* gene lies on the X-chromosome and is known to escape silencing. Several genes related to immune response are also located on the X-chromosome, resulting in a faster and stronger immune defense in females. Additionally, hormones might cause sex differences in COVID-19 outcomes. High concentrations of estrogen have anti-inflammatory properties, thereby alleviating the detrimental effects of the cytokine storm in COVID-19. Lastly, women tend to have less comorbidities than men, due to healthier lifestyles and the protective effects of estrogen.

## COVID-19 and Pregnancy

Pregnancy is a unique physiological condition with changes in the endocrine system (e.g., high levels of cortisol, progesterone and estrogen), cardiovascular system (e.g., increased cardiac output, decreased systemic vascular resistance, higher blood volume) and respiratory system (e.g., swelling of upper respiratory tract, elevated diaphragm, lower total lung capacity, hyperventilation) (229, 230). Considering COVID-19, physiologically elevated basal oxygen consumption levels, a predisposition to developing lung edema and dyspnea, as well as hypercoagulability are all relevant during pregnancy (231–234).

According to a meta-analysis from Di Toro et al. which included 1,100 pregnancies, most pregnant women infected with SARS-CoV-2 had uncomplicated clinical courses with frequent symptoms being fever and cough, pneumonia was prevalent in 89% of the cases (235). They further found an ICU admission rate of 8% and 5 maternal deaths, implying that pregnant women do not face worse outcomes than non-pregnant women. Other studies have also found pregnant COVID-19 patients to have similar clinical characteristics and disease outcomes as non-pregnant women (236, 237). Conversely, higher admission rates to ICU and a higher risk for more severe COVID-19 cases was reported among pregnant compared with non-pregnant patients (238–240). Advanced maternal age, comorbidities such as diabetes mellitus, hypertension, low socioeconomic status as well as obesity have all been identified as possible risk factors for maternal death from COVID-19 (241).

The current pandemic has resulted in the requirement to transform and adapt healthcare services for pregnant women in high-risk groups including women with gestational diabetes mellitus (GDM) or diabetes mellitus. GDM is the most common medical complication of pregnancy and affects 10% of pregnancies globally. Those diagnosed with this condition are at higher risk for a severe COVID-19 infection due to predisposing factors such as hyperglycemia, obesity and hypertension (242). Critically, the most common underlying conditions of pregnant women with SARS-CoV-2 that were hospitalized for severe disease were pre-pregnancy BMI ≥30 kg/m^2^ (41.7%) and diabetes mellitus (Type 2) (12.5%) (243). An interplay of several pathophysiological mechanisms is thought to increase the risk of an unfavorable course and a worse prognosis for patients with GDM. In general, diabetes or insulin resistance reduces T cell function leading to an impaired immune response. This results in the global cellular dysfunction underlying a variety of symptoms associated with diabetes, including a higher risk of respiratory infections (244). Moreover, it has been indicated that some COVID-19 patients develop a diabetes-like syndrome (245). Taken together, these factors could lead to poor pregnancy outcomes including pre-term labor, neonatal admissions to ICU or still birth. Therefore, several healthcare guidelines from the NIH (USA), STIKO/Robert Koch Institute (Germany) and the RCOG (UK) have emphatically expressed the urgency for pregnant women (diabetic and/or obese) to be vaccinated.

Commonly reported neonatal complications are preterm birth, premature rupture of membranes and fetal distress, while intrauterine growth restriction, miscarriage and death are rare events (246, 247). The high cesarean section rate (85%) among COVID-19 positive women is noteworthy, despite COVID-19 not posing a contraindication for vaginal delivery (235). Possibly, improved infection control during labor explains the preference for C-section, however, vaginal fluids were repeatedly tested were negative for SARS-CoV-2, indicating low risk for intrapartum transmission (246, 247).

**Figure 4:**
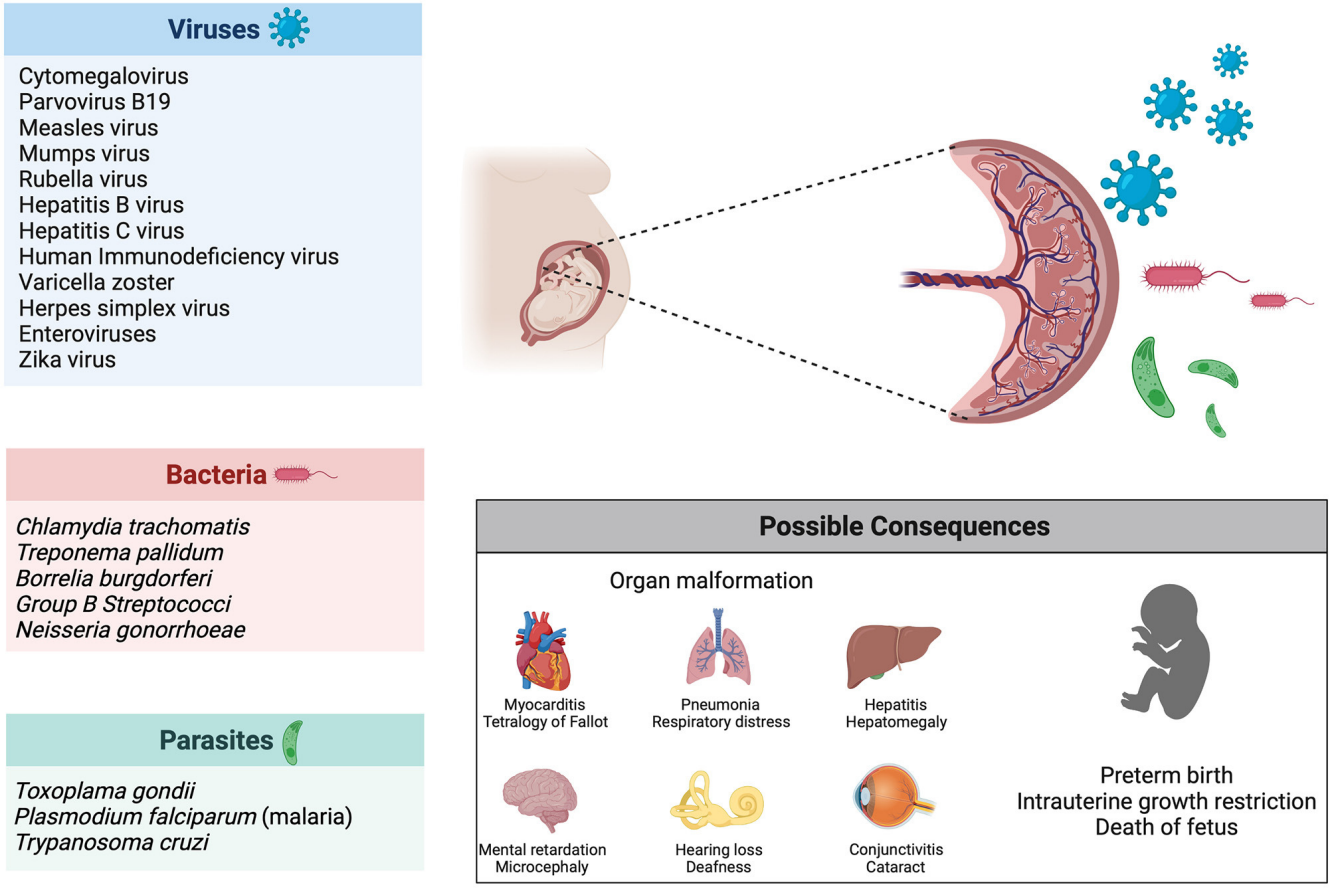
Infection during pregnancy. Several pathogens (viruses, bacteria and parasites) are known to be vertically transmitted during pregnancy. Possible consequences of infection during pregnancy include organ malformations, preterm birth and death of the fetus.

Vertical transmission describes the process of ante-, peri- or postnatal mother-to-child transmission of infectious agents (248). The focus of this review will be on vertical transmission before birth, meaning *in utero* infection of the fetus. Several bacteria, viruses and parasites are known to cause congenital infection, the most common one being the cytomegalovirus (CMV) (249). The consequences of these infections depend on the pathogens, with some causing fetal death (e.g., parvovirus B19, mumps virus, rubella virus) and others leading to malformations or organ defects (e.g., Chlamydia trachomatis, Treponema pallidum, CMV, Toxoplasma gondii) (250). Another determinant of teratogenicity is the time of infection: the rubella virus causes cerebral, cardiac, ophthalmic and auditory defects when infection occurs in the first trimester of pregnancy (during organogenesis), whereas the fetus is most vulnerable to the hemolytic effect of parvovirus B19 and subsequent hydrops fetalis during the second trimester, due to heightened hematopoiesis in the fetal liver (Figure 4) (250, 251).

While SARS-CoV-2 can be passed from mother to infant through respiratory droplets during labor or in the postnatal period, the question of *in utero* transmission remains unresolved (252). Infection rates among neonates born to COVID-19 positive mothers are low (6%), however, cases of early-onset COVID-19 exist with infants testing positive *via* nasopharyngeal swabs within 12 h postpartum (235, 253). Further, antibodies against SARS-CoV-2 identified in newborns shed additional light on the possibility of prenatal vertical transmission (254, 255). In contrast to IgG, which is subject to physiological transplacental transfer and therefore could originate from maternal blood, elevated levels of IgM indicate infection of the fetus *in utero*, as IgM usually does not cross the placental barrier (256). Nevertheless, inflammatory processes can affect the placental barrier and result in altered transfer of immunoglobulin (257). Thus, elevated IgM levels in neonates are no definite proof for *in utero* transmission of SARS-CoV-2.

Furthermore, in a study from Hecht et al. SARS-CoV-2 RNA was detected in the syncytiotrophoblast and cytotrophoblast of placentas from COVID-19 positive mothers (258). This demonstrates that SARS-CoV-2 can infect the placenta, however, it does not definitely confirm vertical transmission. Further, these women were tested (positive for COVID-19) peripartum, limiting the insights into late pregnancy infection.

A potential way for vertical transmission of SARS-CoV-2 during pregnancy is *via* ACE2. As reviewed above, ACE2 is expressed in fetal and maternal tissues during pregnancy and most amply so in the early stages (77–81, 88, 89). Strong co-expression of ACE2 and TMPRSS2, necessary for cleavage of the spike protein, at the maternal-fetal-interface was reported by some studies (78, 259). In contrast, when examining expression patterns of ACE2 and TMPRSS2 in placentas, Pique-Regi et al. only found negligible co-transcription, especially compared to receptors for Zika virus and CMV, both known to cause congenital infections (260). While ACE2 was repeatedly shown to be present in endometrial and placental tissues, research on TMPRSS2 is inconclusive (258, 260, 261). It should be noted that low expression of TMPRSS2 does not necessarily equal low risk of SARS-CoV-2 infection. Other proteases have been suggested to provide an alternate pathway for viral cell entry, such as TMPRSS4, furin or cathepsin L (94). Notably, TMPRSS4 is expressed alongside ACE2 in the endometrium and, akin to ACE2, increases toward the window of implantation (261). Furthermore, TMPRSS4 has the capacity of facilitating cell entry of SARS-CoV-2, thus, making it a candidate for promoting vertical transmission (262).

Another option for *in utero* transmission of SARS-CoV-2 while omitting ACE2 is through infected blood cells. While viremia in COVID-19 exists, only low levels of SARS-CoV-2 RNA are detectable in blood of infected patients (263, 264). When studying the full-term placenta from a COVID-19 positive mother whose newborn was also tested positive, Facchetti et al. showed that viral proteins and RNA were present in numerous fetal and maternal cells (265). Of particular interest is the finding that fetal monocytes were infected with SARS-CoV-2, thus, providing a potential vehicle for vertical transmission.

When interpreting results regarding vertical transmission, it must be considered that most studies solely include mothers tested positive for COVID-19 during the third trimester or peripartum and little attention has been devoted to early pregnancy. Recently, Valdespino-Vázquez et al. examined placental and fetal tissues from a miscarriage during the first trimester of a COVID-19 positive patient (266). They found viral proteins and RNA as well as hyper-inflammation present in both the placenta and the fetal organs (266). As organogenesis occurs in early pregnancy, infection during this crucial time would have detrimental effects on the fetal outcome. Additionally, a case report on a first trimester COVID-19 infection indicated, not only that SARS-CoV-2 persists in the placenta, but it also infected the amniotic fluid and fetal membranes (267). Strikingly, while the mother remained asymptomatic, her unborn succumbed to hydrops fetalis and death (267). Therefore, more research on COVID-19 and its effects on early pregnancy is urgently needed.

Indeed, vertical transmission is not the only way a pathogen can harm an unborn infant. Firstly, SARS-CoV-2 is known to cause placental damage, including maternal and fetal vascular malperfusion, decidual arteriopathy, intervillous thrombi as well as inflammation (e.g., villitis, intervillositis, chorioamnitis) (268–270). Abnormal ACE2 expression caused by SARS-CoV-2 infection in both decidua and placenta could potentially impair key physiological processes, such as placentation and vascularization during pregnancy (271). Hence, dysregulation of RAS could play a critical role in developing preeclampsia-like placental pathology, COVID-19 associated miscarriages and still births. Placental impairment leads to compromised fetal supply of oxygen and nutrients with subsequent complications, such as intrauterine growth restriction or miscarriage, independent of vertical transmission (272). Remarkably, placental pathology exists even in mild or asymptomatic cases of COVID-19 (273, 274).

Aside from locally impacting the placenta, COVID-19 is a systemic disease and the maternal immune response can result in fetal injury (250). Being subjected to an inflammatory milieu, can damage the lungs and brain of the developing fetus (250). Likewise, maternal fever and upper respiratory infection, both characteristics of COVID-19, are linked to congenital heart disease (275). Deleterious effects of the cytokine storm induced by SARS-CoV-2 are not confined to the pregnant woman but could also affect the fetus, possibly resulting in multi-organ failure and ultimately fetal demise (276).

Of equal importance are psychological implications of the ongoing pandemic, such as higher rates of depression and anxiety among pregnant women, which might impact health and well-being of both mother and child (277, 278). Psychological stress is a known risk factor for miscarriage, especially during early pregnancy, which might be linked to elevated cortisol levels (279, 280). Furthermore, the pandemic lead to hampered access to pre- and postnatal care services, possibly contributing to underdiagnosis of complications (281). Interestingly, pregnant women were found to be at lower risk for depressive symptoms in comparison to non-pregnant women and mothers delivering during the COVID-19 pandemic had reduced risk of postpartum-depression than before the pandemic (282, 283). This points to substantial psychological resilience among expectant mothers.

In general, gravid patients present certain challenges to medical treatment. As mentioned above, pregnant women exhibit edema and swelling in the upper respiratory tract, thereby complicating endotracheal intubation (229). Moreover, pregnancy has implications on medication used to treat COVID-19 and vice versa; for example, Favipiravir, an antiviral drug used against COVID-19, should not be administered during pregnancy, and for magnesium sulfate, used for prophylaxis and treatment of preeclampsia, dosage adjustment is required in COVID-19 patients (284, 285).

While vaccination is seen as a promising way to resolve the COVID-19 pandemic, pregnant women were not included in clinical trials and, accordingly, a lack of evidence exists regarding safety and efficacy of COVID-19 vaccines in pregnancy (286). This not only resulted in differing recommendations from national and international organizations regarding vaccination against SARS-CoV-2 during pregnancy, but also in confusion and low acceptance among pregnant women (287, 288). Very recently, evidence about the safety and effectiveness of COVID-19 vaccines for pregnant women is emerging. Preliminary data indicates that vaccination against COVID-19 during pregnancy and lactation is safe regarding maternal side effects, female fertility as well as pregnancy and neonatal outcomes (289, 290). Based on 35,691 volunteers during or shortly before pregnancy, participants did not report any adverse side effects among pregnant women who received mRNA COVID-19 vaccines either from Pfizer/BioNTech or Moderna (290). It was demonstrated for COVID-19 mRNA vaccines that not only an immune response could be elicited in pregnant and lactating women but also that the antibodies could be passed onto their infants through the umbilical cord blood and breast milk (291–294). Given the ongoing pandemic, further research is urgently needed to provide reliable recommendations for the vaccination of pregnant and lactating women.

## Concluding Remarks and Future Perspectives

This review describes the impact of COVID-19 on non-pregnant and pregnant women. Its aim is to explore why the SARS-CoV-2 infection affects men more severely than women and bringing knowledge about genetic, endocrine and exogenous factors together. Different ways of how COVID-19 can harm pregnant mothers and neonates are discussed and currently available data on the possibility of vertical transmission is summarized. This review intends to connect the pathophysiology of COVID-19 to the physiology of pregnancy, decidualization and RAS.

While knowledge on COVID-19 is increasing rapidly, a lack of evidence for the impact on pregnancy remains. Although the currently available data shows that non-pregnant and pregnant women seem to be less affected in terms of severity and mortality, little is known about the long-term effects of viral infection on the fetus. Vertical transmission seems to be rare, but neither is the possibility of *in utero* transmission excluded, nor are the effects of the maternal immune response on the unborn fully understood. Considering the therapeutic obstacles that the pandemic poses on pregnant COVID-19 patients, further research is needed to improve maternal and fetal management.

Notably, a major part of research on COVID-19 and pregnancy focuses explicitly on the third trimester, resulting in a lack of knowledge concerning adverse effects and vertical transmission during early pregnancy. With new SARS-CoV-2 variants emerging, continuous effort is required to shed light on their consequences for pregnancy as well as lactation. Furthermore, pregnant women should not be excluded in future clinical trials for COVID-19 vaccines and therapies. Scientific progress will enable doctors and health workers to provide evidence-based treatments for pregnant women.

## Author Contributions

AL, MS, and JR performed information retrieval and/or analyzed data. MS, YS, and SB provided intellectual input, resources, and funding. AL wrote the original draft of the manuscript and drew the figures. The presented work here is used in MD thesis of AL. All authors reviewed the manuscript and approved of submission.

## Funding

This work was supported by funding to MS intramural funds of Tübingen University the IZKF (2510-0-0) and by the Margarete von Wrangell (MvW 31-7635.41/118/3) habilitation scholarship co-funded by the Ministry of Science, Research and the arts (MWK) of the state of Baden-Württemberg and by the European Social Funds. AL is supported by the Tübingen Medical faculty Interdisziplinäres Promotionskolleg Medizin program (2021-1-02). To YS and MS the Ferring COVID-19 Investigational Grants in Reproductive Medicine and Maternal Health (RMMH) and we also thank the Open Access Publishing Fund of Tuebingen University. The funders played no role in the study design, in the collection, analysis and interpretation of data, in the writing of the report or in the decision to submit the article for publication.

## Conflict of Interest

The authors declare that the research was conducted in the absence of any commercial or financial relationships that could be construed as a potential conflict of interest.

## Publisher's Note

All claims expressed in this article are solely those of the authors and do not necessarily represent those of their affiliated organizations, or those of the publisher, the editors and the reviewers. Any product that may be evaluated in this article, or claim that may be made by its manufacturer, is not guaranteed or endorsed by the publisher.
